# Using Thermomechanical Properties to Reassess Particles’ Dispersion in Nanostructured Polymers: Size vs. Content

**DOI:** 10.3390/polym15183707

**Published:** 2023-09-08

**Authors:** Joel Boaretto, Robinson Carlos Dudley Cruz, Felipe Vannucchi de Camargo, Guilherme Luís Cordeiro, Cristiano Fragassa, Carlos Pérez Bergmann

**Affiliations:** 1 Universidade Federal do Rio Grande do Sul, Porto Alegre 90040-060, Brazil; bergmann@ufrgs.br; 2 Instituto Hercílio Randon, Caxias do Sul 95180-000, Brazil; robinson.cruz@ihr.tec.br (R.C.D.C.); guilherme.cordeiro@ihr.tec.br (G.L.C.); 3 Universidade de Caxias do Sul, Caxias do Sul 95200-000, Brazil; 4 SENAI Institute of Innovation in Polymer Engineering, São Leopoldo 93030-090, Brazil; felipe.camargo@senairs.org.br; 5 Department of Industrial Engineering, University of Bologna, 40126 Bologna, Italy; cristiano.fragassa@unibo.it

**Keywords:** nanocomposites, particle dispersion, mechanical properties, nano-sizing, epoxy polymer

## Abstract

Nanoparticle-filled polymers (i.e., nanocomposites) can exhibit characteristics unattainable by the unfilled polymer, making them attractive to engineer structural composites. However, the transition of particulate fillers from the micron to the nanoscale requires a comprehensive understanding of how particle downsizing influences molecular interactions and organization across multiple length scales, ranging from chemical bonding to microstructural evolution. This work outlines the advancements described in the literature that have become relevant and have shaped today’s understanding of the processing–structure–property relationships in polymer nanocomposites. The main inorganic and organic particles that have been incorporated into polymers are examined first. The commonly practiced methods for nanoparticle incorporation are then highlighted. The development in mechanical properties—such as tensile strength, storage modulus and glass transition temperature—in the selected epoxy matrix nanocomposites described in the literature was specifically reviewed and discussed. The significant effect of particle content, dispersion, size, and mean free path on thermomechanical properties, commonly expressed as a function of weight percentage (wt.%) of added particles, was found to be better explained as a function of particle crowding (number of particles and distance among them). From this work, it was possible to conclude that the dramatic effect of particle size for the same tiny amount of very small and well-dispersed particles brings evidence that particle size and the particle weight content should be downscaled together.

## 1. Introduction

The field of materials science has seen one of the most interesting developments in the emergence of nanostructured polymers. These materials are characterized by their unique properties, which are largely due to the dispersion, size and content of the nanoparticles that they contain. The addition of nanoparticles promotes significant changes to the mechanical, thermal, electrical and optical properties of polymers, making them ideal for a wide range of applications, from biomedical engineering to energy storage [1,2].

Fibrous reinforcements in a polymeric matrix also offer several advantages, making them a popular choice in composite materials. The most commonly used fibers are glass, carbon, aramid and basalt, which are selected to provide the necessary stiffness and strength to the composite, depending on the fiber’s orientation, length, volume and interfacial properties. As the polymeric matrix is responsible for the stress transfer at a micromechanical level, and thus for the performance required, its mechanical properties, because they are affected by environmental conditions, such as temperature, humidity, radiation and exposure to chemicals, are of utmost importance and can hinder the final component functionality [2,3,4].

The cost of polymers increases with their degree of engineering. Bearing in mind the importance of the property-to-cost balance to produce viable and scalable composites, the need to finely tune polymers has been looked at from the perspective of tailored-made nanostructured materials by several investigations in the last decades, shedding light on a class of materials known as nanocomposites, disseminated by the Toyota research group and literally opening a new dimension in materials science [5,6,7].

This development has emphasized the importance of factors like particles’ specific surface area and mean free path, showing the intimate relationship among the amount of added nanoparticles, faultless dispersions and the resulting properties. This suggests a good economic balance and unveils an encouraging scenario for tailor-made applications and high-performance materials.

Inspired by the above, this article reviews the incorporation of distinct particles in epoxy matrices, analyzing how important parameters such as dispersion methods, particle size and particle content affect the properties of nanostructured polymers. The particles mostly used in particle-filled polymers are described first. Next, widely practiced techniques for incorporating nanomaterials while preventing agglomeration are summarized, emphasizing the processing difficulties. A discussion follows on how particle downsizing influences the particle–epoxy mechanical properties, providing a different perspective on processing–structure–property relationships.

## 2. Major Particle Fillers

In light of the distinct features of the numerous types of fillers described in the literature, only those judged more relevant are discussed below, along with advances in the respective field.

### 2.1. Inorganic Nanofillers

Inorganic nanofillers present some significant advantages over most organic ones. Except for cellulose, inorganics tend to be much more scalable in production than carbon nanotubes (CNTs) or graphene, for instance, while still providing interesting property enhancements to polymer composites. Among the inorganic fillers, titanium dioxide (titania, TiO_2_), silicon dioxide (silica, SiO_2_) and zinc oxide (ZnO) have been widely used and thoroughly studied [8].

#### 2.1.1. Titanium Dioxide

TiO_2_ (titania) is a natural oxide found in three allotropic forms: rutile, anatase (both tetragonal) and brookite (orthorhombic). Applications make use of mostly rutile and anatase and are, as such, categorized into two groups.

Rutile is brown-red colored and has the highest density, with antibacterial and UV-absorbent properties. For these reasons, applications include the military industry, cosmetics, soap, sunscreen, toothpaste, cigarette paper and paints to coat electronics, toys, furniture and packages [9]. On the other hand, anatase has a lower density and appears in different colors (brown, yellow, orange, green and blue). Its applications include solar cells, catalysts, sensors and battery components. An interesting feature of anatase is its brightness, which enhances the whiteness of paper and removes the greasy-look of resin-coated fibers. It is also used in rubber formulation due to its anti-aging properties [9].

As a filler, nano-TiO_2_ is scalable and extensively used in various industries for multiple purposes (mostly photocatalysis, anti-corrosion and chemical stability), ranging from antibacterial materials, anti-fogging mirrors and electronics to sunscreens and cosmetics [9]. Particles may be synthesized by numerous methods, with distinct shapes, sizes and properties [10].

When incorporated in polymer matrices, nano-TiO_2_ is expected to enhance the mechanical, electrical, optical and rheological properties [11]. It may be incorporated either as particles in the matrix [12] or as fiber coating [13]. The latter is particularly interesting for natural fibers given the offered barrier properties, thus substantially decreasing the water absorption capability of the fibers and protecting the composite from humidity-related aging.

From the perspective of the influence on the mechanical properties of composites, Hunain et al. [14], for example, demonstrated the beneficial effect of titanium dioxide on the dynamic mechanical properties of fiber-reinforced polymer composites. The increase in fatigue resistance could be attributed to the influence of the filler simultaneously on the matrix, making it tougher and more rigid over the interphase regions, ensuring a higher adhesion among the fibers and matrix. As another illustration, Al-Zubaydi et al. [15] showed that the dispersion of this filler in an epoxy matrix enhanced its resistance to wear, which, allied with the known chemical and impact resistances of the composite, makes it an interesting material for anti-skid flooring applications.

#### 2.1.2. Silicon Dioxide

SiO_2_ (silica) may be found in various crystalline and amorphous structures, with low thermal conductivity and high chemical inertness towards most substances.

The compound is abundant throughout the planet and originates from many sources, amongst which sand is the most common. Due to its availability and properties, silica is widely used in industry in fields such as civil construction (mostly in Portland cement concretes), food, pharmaceuticals, cosmetics, paints, inks, rubbers, glass, aerospace, automotive and electronics (semiconductors) [16].

As a filler, nano-SiO_2_ is used for its scalability and ability to increase the polymer’s mechanical, thermal and electrical properties [17]. It has proven to be quite effective in decreasing the frictional wear of polyether–ether–ketone (PEEK) composites [18] by reducing its friction coefficient and increasing the UV resistance of epoxy, both as hydrophilic or hydrophobic nanosilica [19]. It has also been found that both DC resistivity and the dielectric breakdown strength improve in epoxy-silica nanocomposites [20], which is supposedly due to the effectiveness of the organic–inorganic interface, opening possibilities for the development of cutting-edge thermoset composites.

#### 2.1.3. Zinc Oxide

ZnO is a multifunctional filler with a particular set of features, including a high refractive index, high thermal conductivity, antibacterial properties and high UV-shielding, which makes it a strong candidate for reinforcing translucent and UV-resistant composites [21]. These are specifically attainable at a filler content lower than usual (0.03 wt.%). Nano-ZnO epoxy-based coatings can promote surface charge dissipation on insulators in DC gas-insulated systems [22] while also being anti-corrosive due to their hydrophobic nature [23]. Due to its amenable intrinsic and processing characteristics, ZnO is generally preferred over other possible competitors [24].

Pure ZnO is a white powder with hexagonal wurtzite and the cubic zincblende structure [25,26], naturally occurring as the mineral zincite, usually containing manganese and other impurities that confer a yellow to red color [26]. Zinc oxide is an amphoteric oxide and is nearly insoluble in water; however, it will dissolve in the majority of acids [26].

The applications of zinc oxide powder are numerous, and most exploit the reactivity of the oxide as a precursor to other zinc compounds. Currently, it is added to materials and products, including plastics, ceramics, glass, cement [26], rubber, lubricants [27], baby powder and creams against diaper rashes, calamine cream, anti-dandruff shampoos, antiseptic ointments [28], pigments for paints [29], and Li-ion battery and supercapacitors [30] to name some.

#### 2.1.4. Clays

Clays are natural silicate minerals that are divided into several groups according to their composition and particle morphology, such as kaolinite, illite, smectite and vermiculite. Most have a sheet-like morphology that tends to form stacked structures; however, some can roll over and form tubular structures (e.g., halloysite, from the kaolinite group). Those stacked structures can be spread-out into nearly individual high-aspect ratio sheets of nearly 1 nm in thickness and around 100 nm in planar dimensions, as explained by Saba et al. [6] in a very comprehensive review. Similar to CNTs, halloysite nanotubes (HNT) may also be single- or multi-walled [30] but are much cheaper, more abundant and sustainable when compared to CNTs. The layered architecture allows water adsorption/desorption and enables cation exchange, which is vital to define the effectiveness of surface modifications. This can convey an organophilic character to these particles, making them suitable for dispersion in hydrophobic organic polymeric matrices [31].

When incorporated into polymeric matrices, these nanofillers, as others, are only a minor proportion of the composite and still provide enhancements in physical, thermal and mechanical properties, such as permeability, flammability and tensile toughness, respectively [25,32,33,34]. These improvements enable the use of the composites in diversified applications, such as flame-retardant plastics, high-clarity films [30], dental [35] and drug delivery systems [36].

### 2.2. Organic Nanofillers

#### 2.2.1. Graphene, Graphene Oxide and Graphene Nanoplatelets

Graphene is a single-layer, two-dimensional sheet of sp^2^-hybridized carbon atoms [37] that has earned a reputation for being capable of enhancing the properties of reinforced polymers, from fracture strength [38] to electric [39] and thermal [40] properties. These improvements are possible thanks to characteristics such as a high surface area and aspect ratio [41], good interfacial adhesion to thermosets, such as epoxy [38], and its high mechanical strength. The applications of this filler are very diversified and include tissue engineering, drug delivery systems [42], magnetic resonance imaging [43] and cancer therapy [44].

The main behavioral difference between graphene oxide (GO) and graphene is the fact that the first is hydrophilic and the latter hydrophobic. This change in polarity can be explained by the presence of oxygen bonded through the sp^2^ in GO, and it enables the dispersion of GO in aqueous solutions, enhancing its applicability to polymeric composites, as well as functionalization [45].

Graphene nanoplatelets (GNPs) are small sheets of graphene and, as such, can be effectively embedded in polymeric matrices, enabling many applications due to the increase in thermal and electrical conductivities and a reduction in the porosity of polymers [46].

Nevertheless, it is important to highlight that graphene is not yet applied on a large scale for a few reasons. Firstly, achieving maximum purity in its production process seems to be quite challenging [38]. Moreover, the production cost is currently a major barrier to scalability [47]. Finally, storage, transportation and health and safety issues are also current concerning topics [48].

#### 2.2.2. Carbon Nanotubes

Carbon nanotubes (CNTs) are basically rolled-up graphene sheets. These CNTs might have one and two or more concentric layers, respectively, called single-walled (SWCNT) or multi-walled (MWCNT). Although the property enhancement from the inherent carbon interatomic bonds is retained, reinforcement by CNTs is unidirectional, unlike the 2D graphene sheets. As such, the reinforcement is most advantageous if the CNTs are aligned in the direction of the applied load. The elastic strain energies of CNTs, however, tend to be affected due to the curvature of the carbon bonds in the tubular shape [46]. Biomimetic hierarchical composites using fibers and CNTs have also been investigated [49,50,51,52], with inconclusive results. Aside from the mechanical improvements that this filler confers to composites, it is important to underline their influence over thermal and electrical properties [51], enabling applications in the fields of nanoelectronic devices, medicine and defense [41,42,43,44,45,46,47].

#### 2.2.3. Nanocellulose

Cellulosic fillers are amongst the most abundant, sustainable, biodegradable and cheap nanofillers. Its main nanometric forms are cellulose nanocrystals (CNC) and nanofibrillated cellulose (NFC). Cellulose nanocrystals, for instance, are obtained from cellulosic fibers by extracting nanofibrils, removing hemicellulose and lignin through bleaching, and then removing the amorphous regions linking each CNC by acid hydrolysis [53].

Recent research has shown that the addition of CNC to polymeric coatings significantly inhibits corrosion in mild steel, increases the elastic modulus and impact strength in rubber vulcanization and may also be effective in producing tough and resilient polyacrylamide-matrix hydrogels [54,55,56].

NFCs are the structures immediately above CNCs, being basically nanometric in diameter and micrometric in length and including the amorphous regions between two CNCs. Because it is longer and more flexible than CNCs, NFCs have some advantages, such as their longer strain to fracture and higher surface area, which enables them to provide a good adhesion with the polymeric matrix. An example of a recent application promoted by this feature is the use of NFC aerogel for developing wearable strain sensors [57].

## 3. Dispersion Methods of Nanoparticles in Polymeric Matrices

Physical processing methods for dispersing nanoparticles in a polymeric matrix are currently of significant interest. Melt-blending and solvent processing, assisted by high-shear mixing or sonication, have been favored in important research contributions due to simplicity and compatibility with standard industrial techniques. Melt blending is one of the most economical methods for fabricating nanocomposites and is the easiest to scale up for a wide range of polymeric composites [47,58,59,60,61,62,63,64,65,66,67,68,69,70]. The method obviously requires temperatures typically higher than the melting point of the polymeric matrix.

The dispersion of the nanoparticle fillers within the polymeric matrix can be achieved by applying high shear forces using, for example, single- or twin-screw extruders or compounders, which help to break apart the filler agglomerates. Its simplicity makes it attractive for industrial-scale processes; however, many parameters (rotation speed of the twin-screw, temperature, high-shear mixers, hydrodynamics, etc.) must still be fine-tuned to optimize the results. However, it has also been observed that adhesion between the matrix and filler is not favored in melt processing, as compared to the very strong interaction between individual nanoparticles [47]. While melt blending of hard-to-wet, untreated carbon nanotubes (CNTs) and graphene has shown modest improvement in dispersion, the melt mixing of montmorillonite clay with polymer has met with great success. The chemical functional groups at the silicate surface of the individual clay platelets might explain the observed greater affinity that results in better adhesion with the matrix.

On the other hand, solvent processing coupled with mechanical mixing or sonication is also suitable for obtaining nanocomposites [9,11,47,60,63,71,72,73,74,75,76,77,78,79,80,81]. The technique involves dispersing the nanoparticles in a polymer that is firstly dissolved in a compatible solvent. The breaking up of particle agglomerates is promoted by either a turbulent flow or the formation of cavitation bubbles. The major disadvantage, of course, is that the method is limited to polymeric materials that can be easily dissolved. In addition, the solvents used in the sonication process need to have low viscosity (e.g., acetone, distilled water, ethanol [63]).

Although these physical methods are supposed to be envisioned to benefit industrial processing, it is worth mentioning that most of the studies pointed out that a fully homogeneous dispersion of nanoparticles is difficult to attain unless a rather low content of nanoparticles (typically < 1.0 wt.%) is used. Furthermore, the mixing or processing conditions may need to be tailored by introducing compatibilizers or surfactants to improve the quality of filler dispersion. The recent review by Boon et al. [80] suggested that ionic surfactants should be used for CNTs in aqueous mixtures, whereas nonionic surfactants are preferred for mixtures with organic solvents. Some of the ionic surfactants used with carbon nanotubes include sodium dodecyl sulfate (SDS), dodecyl-benzene sodium sulfonate (NaDDBS) and polyvinyl pyrrolidone (PVP), while nonionic ones include polyoxyethylene-8-lauryl and Tergitol NP-7 [80,81,82]. 

To improve the interactions between nanofillers and a matrix, chemical methods are needed. Functionalization has so far been preferred [9,11,22,45,51,52,68,78,83,84,85,86,87,88,89,90]. The concept relies on the attachment of a specific functional group at the filler’s surface, which promotes uniform polymer–particle suspensions and, after curing, restrains the nanofiller within the matrix. Functionalization can also prevent re-agglomeration by generating steric repulsion among chemical groups with larger molecules/long chains.

Further immobilization can be obtained if the particles are dispersed within the monomers prior to polymerization [47,58,60,63,65,68,78,79,91,92,93,94,95,96]. One of the advantages of this technique is that it allows the grafting of polymeric molecules onto the surface of the fillers, which also leads to a better dispersion of hard-to-wet nanomaterials. 

In contrast to the physical methods, such an in-situ polymerization route offers the added advantage of higher nanoparticle loads.

The effects of the nanoparticle dispersion method on the physical and chemical structures of composites are widely reported in the literature. Observed changes in glass transition temperatures (T_g_), for instance, were attributed to a loss in the flexibility of polymer chain segments resulting from the particle–matrix interaction, which relies on well-dispersed nanoparticles within the matrix. The selected results are shown in Table 1.

Combining nanoparticles with other nanofillers to make hybrid composites has been shown to help the preparation of polymer–particle mixtures [84,91,98]. Coupled with a chemical or a mechanical method, this technique emphasizes the potential of a percolated network of hybrid filling nanomaterials, leading to a significant improvement in nanoparticle dispersion within the polymeric matrix [99]. When compared to functionalization, hybridization typically comes at a cost; however, it eases the fabrication process and has become a promising way to counterbalance some of the manufacturing challenges. 

From a more applied perspective, the applicability of these concepts to the development of structural fiber-reinforced polymeric nanocomposites still needs to be assessed and demonstrated.

## 4. Particle Effects on Composite Thermomechanical Properties

Aside from the complexities summarized above for the particle incorporation processes, published research not only shows that the properties of nanostructured polymers may be altered by the addition of nanofillers but also that those changes depend on the particle type, size and content [87,98]. The major trends on this issue are described in what follows.

To enable a consistent comparison among the variables involved, the polymer matrix was chosen for the epoxy resin, the four most cited nanoparticles, namely TiO_2_, SiO_2_, GO and GNP, were selected, and three properties, namely tensile strength (TS), storage (elastic) modulus (E’) and glass transition temperature (T_g_), were studied.

The titania (TiO_2_) particles are, indeed, the most commonly used particles found in the literature, with more abundant data and several industrial applications. Similarly, epoxy DGEBA is the most common polymeric matrix considered in these studies, given its wide usage in industry, easy access to and ability to disperse particles within low-viscosity media [11].

The shift in the selected properties as a function of particle type, size and content (wt.%) was referred to (linearly normalized by) that of the corresponding epoxy matrix alone and expressed as a percentage (Δ%) in order to level the information extracted from the literature. Table 2 summarizes the tensile strength (TS) data collected. In a similar way, Table 3 presents the data collected for the corresponding storage (elastic) modulus (E’, related to the loss, or viscous, modulus E” by tan δ = E”/E’) and the glass transition temperature (T_g_) for the same nanoparticles and the epoxy polymer matrix. 

In order to enable a concise yet representative analysis, the discussion that follows was narrowed down to titanium dioxide (TiO_2_) particles. 

To normalize the information gathered from different authors, the influence of the particle size and weight content on properties was described in terms of the surface area. The specific surface area (SSA), commonly expressed in m^2^/g, can be calculated from the particle size, assuming a spherical shape for the oxide particles (Equation (1)) [134].

(1) $${SSA}_{sphere}=6/\rho \cdot d$$

In Equation (1), ρ and d represent the particle’s density and diameter, respectively. For TiO_2_, the density considered was 4.23 g/cm^3^ [135].

### 4.1. Effect on Tensile Strength

**Figure 1:**
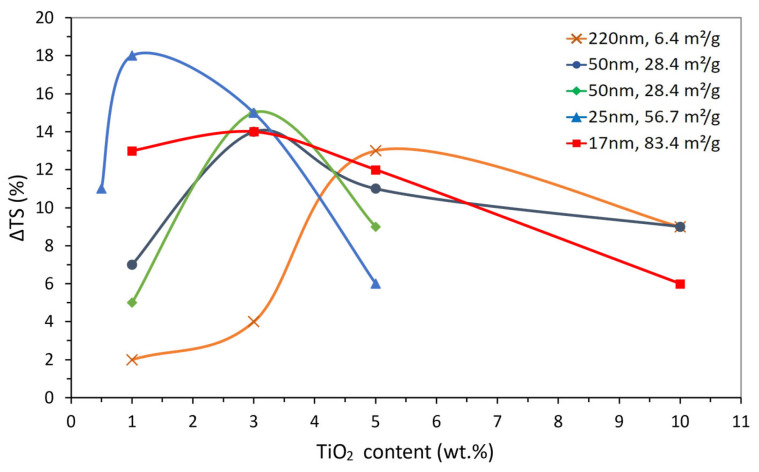
Tensile strength variation (ΔTS) for TiO_2_-filled epoxy composites as a function of TiO_2_ weight content for the particle size and SSA values shown.

Figure 1 was constructed from the selected data listed earlier in Table 2, which is plotted in the way usually found in the literature, i.e., as a function of the weight content of the added particles. As there is no known mathematical relationship (i.e., dependency law) between the tensile strength and added particle content, data points were simply linked by smoothed lines to help the discussion.

Figure 1 shows that, regardless of the TiO_2_ content and SSA, the composite’s TS was always higher than that of the corresponding epoxy matrix. As a common trend, Figure 1 also shows that there is an optimal particle content that results in a maximum TS gain. Above the optimal content value, the TS gain drops, apparently due to ineffective particle dispersion: the higher the TiO_2_ content, the harder it is to avoid particle agglomeration. More importantly, it appears that the optimal particle content tends to decrease as the corresponding SSA increases, i.e., higher TS gains might be obtained with lower contents of smaller particles (high SSA).

Figure 1 also highlights several inconsistencies, pointed out as follows. The gain in TS was markedly influenced by the SSA for any given particle weight content. This could be attributed to the influence of the dispersion method on ΔTS, which can be illustrated at a fixed TiO_2_ content of 1 wt.%. As the SSA increased from 6.4 to 28.4 m^2^/g, ΔTS increased from ~2 to ~7%. On the contrary, when the SSA increased from 28.4 to 56.7 and then to 83.4 m^2^/g, the gain in TS increased from ~7 to ~18 and then fell to ~13%. This drop in ΔTS could suggest the existence of an optimal particle size (or SSA) for the best property gain using a particular added particle weight content. However, it might also be attributed to the structural changes due to differences in particle dispersion, given that, for 56.7 m^2^/g, TiO_2_ particles were incorporated into the epoxy by sonication [102] whereas, for 83.4 m^2^/g, by using a moderate-speed mixing method [100]. As discussed in Section 3, sonication induces the generation of collapsible cavitation bubbles, leading to a more effective dispersion.

The doubtful confidence on the effect of added particle content can be illustrated by referring to the two different authors (Table 2, [100,101]) that explored similar 50 nm TiO_2_ particles (SSA of 28.4 m^2^/g) seeking to improve the tensile strength performance but used a different added particle content range. As seen in Figure 1, the resulting curves do not seem to match each other, even for the common particle content.

Thus, when specifically observing the particle influence on the structuring process, particle size (expressed as SSA) certainly is an important variable; however, particle crowding (namely expressed as the mean free path between them, g, and the particle number, NP) is not suitably translated by weight content, according to the majority representation shown in works with nanocomposites [136,137,138,139].

The number of particles, NP, can be calculated (again assuming identical spherical particles) through the volume fraction, *V_p_*, from the corresponding weight fraction, as seen in Equations (2) and [140].

(2) $${V_{p}=\frac{\rho_{m}\;w_{p}}{\rho_{p}\left(1-w_{p}\right)+{\;\rho }_{m}w_{p}}}_{\;}$$

(3) $$NP=V_{p}/V_{sp}\;$$

In Equation (2), ρ is the density, *w* is the weight fraction, and subscripts *m* and *p* refer to the matrix and particle, respectively. In Equation (3), *V_sp_* is the volume of each particle.

**Figure 2:**
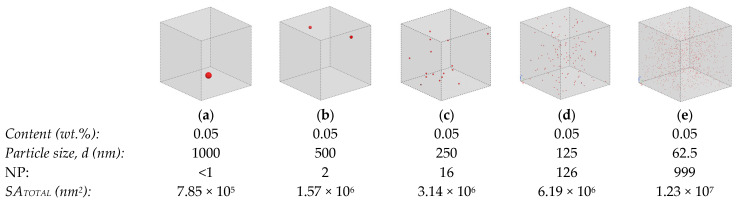
Dispersion of 0.05 wt.% TiO_2_ within a 10 × 10 × 10 μm^3^ volume illustrating particle crowding as a function of TiO_2_ particle diameter (software: Digimat^®^ with Random Fiber Placement algorithm). (**a**–**e**) show the particle number progressively increasing as much as the particle diameter reduces, even though the amount of mass has remained unchanged.

Figure 2 was constructed using the commercial software Digimat^®^ (Version 2021-3 by Hexagon) and the Random Fiber Placement algorithm (the algorithm places the particles at random positions within the cube until the specified volumetric fraction is reached), illustrates the crowding of 0.05 wt.% TiO_2_ particles in a 10 × 10 × 10 µm^3^ volume as a function of the particle size. As the particle diameter is halved, the SSA duplicates (from Equation (1)); however, NP increases sharply (multiplies by 2^3^). As seen in Figure 2, the same small weight content of well-dispersed TiO_2_ particles could result in a homogeneous composite with properties that improve when the particle size decreases (i.e., SSA increases). However, Figure 2 clearly shows that downsizing can result in an excessive number of particles, whose crowding will bring about dispersion difficulties (e.g., agglomeration) and consequently, hindered properties.

**Figure 3:**
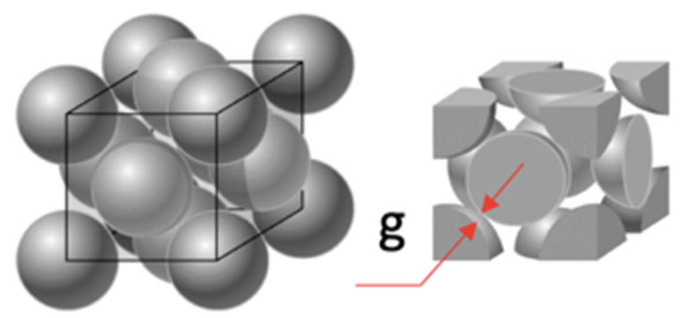
Schematic representation of the FCC particle packing, showing how to calculate the mean free path, g (adapted from LibreTexts^TM^ [33]).

Particle crowding can be assessed through the mean free path among particles, i.e., the distance between the surface of the closest particles. To calculate the mean free path, a specific particle arrangement needs to be assumed. To this aim, the concept of atomic packing factor is what most resembles the studied conditions. The densest particle packing, corresponding to the face-centered cubic arrangement (74 vol.%), was chosen (Figure 3), assuming, yet again, spherical particles. This would be the stringiest particle crowding condition, i.e., in actual homogeneous, well-dispersed composites, g would present higher values.

Figure 3 shows the arrangement of particles for the face-centered cubic packing and highlights the mean free path (g) as the distance between the closest spheres, which can be calculated by Equation (4) [134]. In Equation (4), *d* is the particle diameter, and *V_p_* is the volume fraction of particles.

(4) $$g_{FCC}=\left(\frac{\sqrt{2}}{4}\;\sqrt[{3}]{\frac{16\pi }{3V_{p}}}-1\right)d$$

The number of particles for a given mass percentage is directly proportional to its diameter, d. The smaller the diameter, the greater the number of particles and, consequently, the smaller the distance g among them. In the case of this review, the values of mass percentages and particle diameters were given by the authors, requiring the transformation of this information to a volumetric percentage and, later, the relative number of particles.

**Figure 4:**
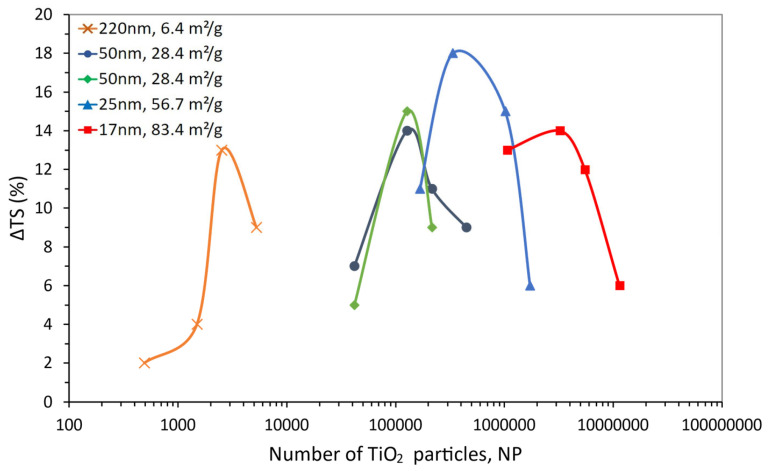
Tensile strength variation (ΔTS) for TiO_2_-filled epoxy composites as a function of the number of TiO_2_ particles (NP) for the particle size and SSA values shown.

Thus, smaller particles can stand much closer than larger particles (g is directly proportional to the particle size). Hence, there is a negative power law dependency between g and the number of particles, NP, or an inverse linear relationship between log (g) and log (NP). The values calculated for the number of particles, mean free path and specific surface area for the TiO_2_ particles used earlier in Table 2 and Figure 1 are presented in Table 4 and expressed graphically in Figure 4. 

Figure 4 allows a clearer analysis of the effect of TiO_2_ particles on epoxy compared to that shown in Figure 1. The dependency of the tensile strength variation with the particle number enables a distinct separation of the ΔTS maxima and brings to evidence the influence of the surface area (i.e., particle size and content), which was not apparent in Figure 1.

**Figure 5:**
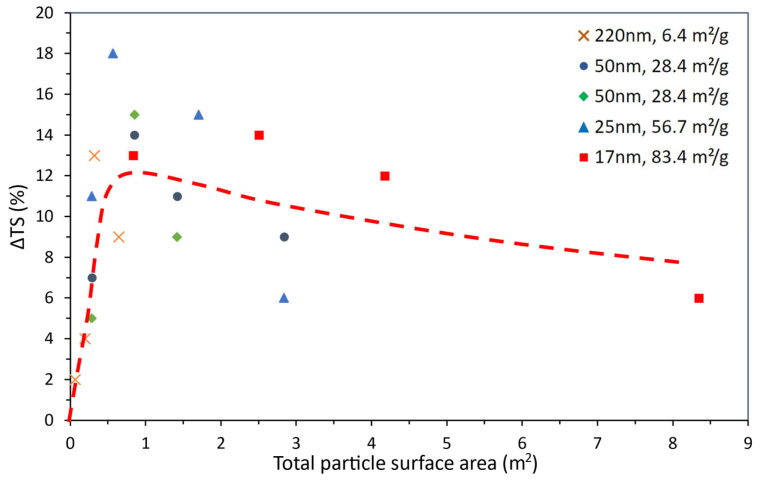
Tensile strength variation (ΔTS) for TiO_2_-filled epoxy composites as a function of the TiO_2_ particles’ total surface area. The dashed line represents the general trend among data points.

The first point worth mentioning is that when the property is plotted as a function of NP, a better overlap can be seen for the two 28.4 m^2^/g curves, despite the particular processing parameters considered in each study [100,101], which the previous and traditional form of portraying data (in wt.%, as seen in Figure 1) did not show. Although the maximum on the property curves was seen before, the representation in Figure 4 clearly separates such maxima, bringing to evidence their dependence on the specific surface area. In other words, for comparatively large particles (i.e., low SSA), the number of particles required to reach the maximum is low; as the particles are downsized (i.e., increasing SSA), the NP for the maximum also increases. Moreover, Figure 4 shows that the maxima on ΔTS are clearly higher for the lower particle sizes while suggesting that there might be an optimal particle size (or SSA) for the maximum property gain, as envisioned earlier in Figure 1. When Figure 1 and Figure 4 are analyzed together, as the particle size decreases, the weight content needed for the maximum property gain also decreases; however, the number of particles increases, i.e., the maxima move backwards in Figure 1 and forwards in Figure 4. This is a direct consequence of the correlation between volume and weight through density for any given type of material, yet it also hints at the possibility that the property gain might be dependent on the particles’ total surface area. In other words, smaller particles would be needed in lower-weight contents to reach a comparable property gain. Figure 5 depicts such an exercise, as it gathers all data in Table 4 by plotting ΔTS as a function of the particles’ total surface area (i.e., weight content × SSA). Despite the scatter in the data, the dashed line in Figure 5 represents the general trend among the data points (moving average trend line), suggesting that the location of the maxima observed earlier in Figure 1 and Figure 4 might be just fortuitous.

**Figure 6:**
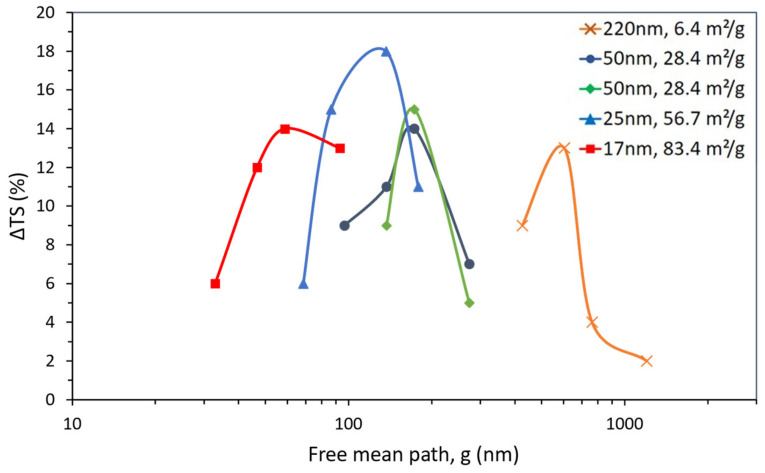
Tensile strength variation (ΔTS) for TiO_2_-filled epoxy composites as a function of the mean free path among TiO_2_ particles (g) for the particle size and SSA values shown.

Thus, it is desirable to find an alternative way to access the property’s underlying mechanism. When the property gain is plotted as a function of g (Figure 6), a trend similar to that for NP can be observed, i.e., the distance among larger particles (with a lower SSA) for the best property improvement is higher than that for smaller particles (with a higher SSA).

**Figure 7:**
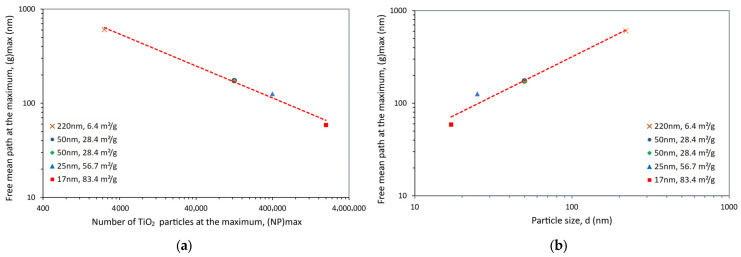
Power law relationship observed at the maximum tensile strength gain for TiO_2_-filled epoxy composites between (**a**) particle number, NP, and mean free path, g, and (**b**) particle diameter, d, and mean free path, g.

The expected relationship between g and NP at the property maximum, i.e., the negative power law relationship (or the linear relationship between log NP and log g), can be observed in Figure 7a. More important, however, is the positive power law relationship between g and d at the property maximum, which can be seen in Figure 7b. If the strengthening of the polymer matrix relies on its texturing around the filler particles, it would have been expected that the maximum benefit would correspond to a particular optimum distance among particles, i.e., to a common g value. That is not what Figure 7b shows. Indeed, Figure 7b suggests that the “affected” matrix volume surrounding each particle depends on the particle size and increases with it. At the point of maximum gain, larger particles will be surrounded by a thicker affected matrix layer than smaller particles and will then need to stand further apart from each other.

Having no direct experimental access to the values of g, it is important to determine if the enhanced contrast provided by this alternative form of representation also works for other properties. 

### 4.2. Effect on Dynamic Mechanical Properties

By the fact that it is a viscoelastic material, the nanocomposite needs evaluations both in the viscous phase and elastic. Because it allows the evaluation of properties in the temperature domain and because it offers mechanical excitations compatible with the scale of the new material (nanometers), a large number of researchers [141,142,143,144,145] use DMA to determine the storage modulus parameters (E’), loss modulus (E”) and glass transition temperature (T_g_). Therefore, extending the reasoning just discussed for the tensile strength, the following analysis considers the results for E’ and T_g_, obtained from dynamic mechanical analysis tests (DMA). Table 5 presents data for the TiO_2_ particles selected from those listed earlier in Table 3. 

#### 4.2.1. Storage Modulus

The first point to note is that there should not be negative gains in the composite modulus. The addition of inorganic (crystalline) particles to a softer polymeric matrix should always result in stiffness gain. Such a discrepancy is frequently attributed to processing difficulties (e.g., bad dispersion, lack of adhesion), meaning that the added particles behave as defects or impurities rather than playing the expected role of strengthening aids. Nevertheless, available data as those listed in Table 5, are scarce and, bearing the above in mind, they were used in the discussion, all the same.

**Figure 8:**
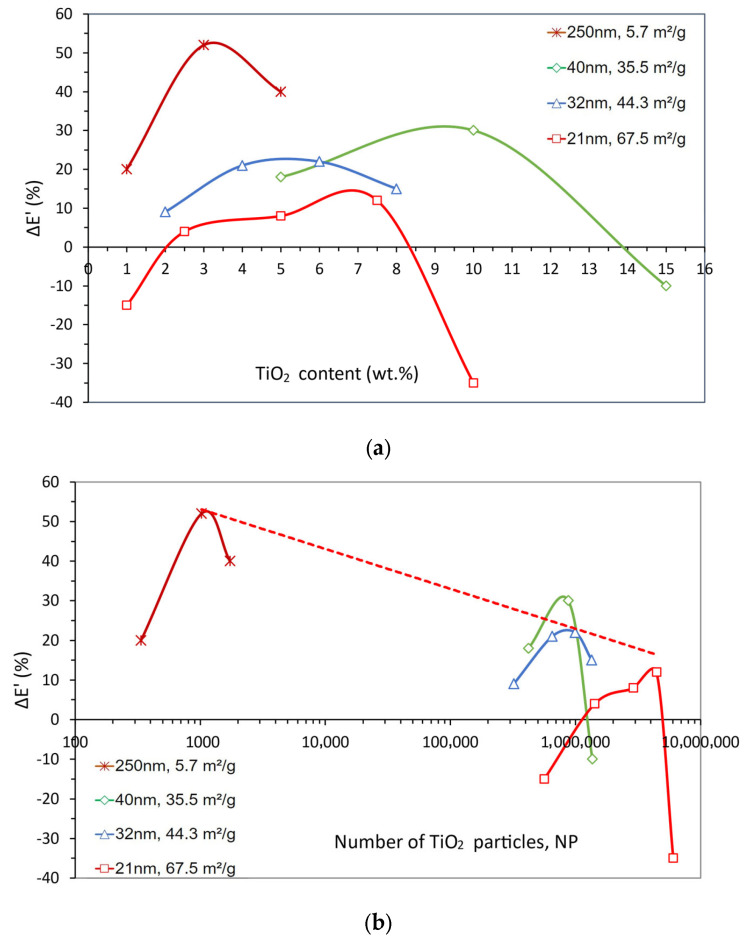
Storage modulus variation (Δ*E*’) for TiO_2_-filled epoxy composites as a function of (**a**) TiO_2_ weight content and (**b**) the number of TiO_2_ particles for the particle size and SSA values shown. The dashed line in (**b**) represents the common straight line among the Δ*E*’ maxima.

The composites’ storage modulus variations (ΔE’) as a function of TiO_2_ weight content and number of particles are presented in Figure 8a,b, respectively. As seen for the tensile strength analysis, both representations show that the storage modulus gain (∆E’) for each different SSA increases up to a maximum value and then decreases.

Figure 8a shows that the highest gain (52%) was reached with small contents of large particles (small SSA, 5.7 m^2^/g). Composites with smaller particles (higher SSA) tend to present their best property at higher particle contents. This effect is clear for the SSA values of 35.5 m^2^/g and 67.5 m^2^/g, where the variation in storage modulus is negative, i.e., despite the awkwardness of this concept, it means that the composite is worse than the epoxy alone.

This effect becomes much clearer from the perspective of the number of particles, shown in Figure 8b. For instance, considering the particles with 5.7 m^2^/g SSA, a small number of particles results in a high gain for this composite. The others, prepared with smaller particles (i.e., higher SSA), even if added in the same weight content, need a much higher number of particles, with the dispersion difficulties that entails, to reach their best increment in modulus.

**Figure 9:**
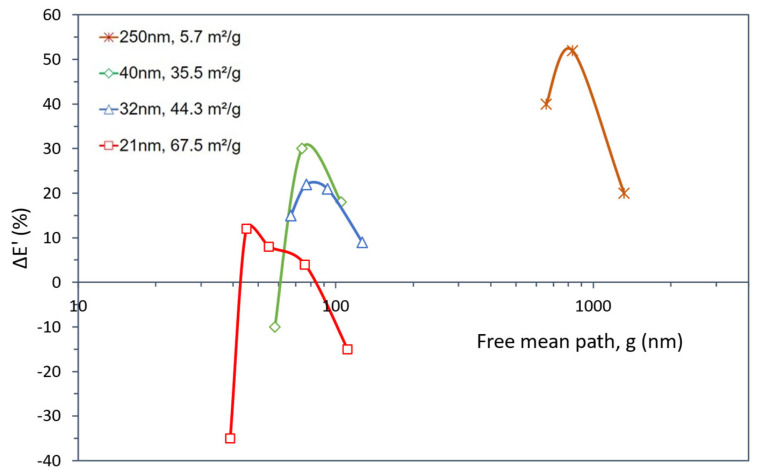
Storage modulus variation (Δ*E*’) for TiO_2_-filled epoxy composites as a function of the mean free path among TiO_2_ particles (g) for the particle size and SSA values shown.

Still, in Figure 8b, another trend can be observed, which is that the peaks of the four curves nearly fall on a straight line, also shown in the graph, suggesting an inverse proportionality between the modulus gain and the log (NP). This is to say that, despite the size gap between the micro and nano size ranges in the data, a high number of particles with high specific surface areas do not provide significant stiffness gains. As it is, the reported low stiffness improvements (and perhaps also the negative gains mentioned above), generally attributed to high SSA, hence poor dispersion, might be the result of particle crowding (particles too close together), which can be evaluated in terms of the mean free path, g, as shown in Figure 9.

Figure 9 clearly explains the better performance of the 5.7 m^2^/g composite because the average distance among its particles is noticeably higher than in the others. In addition, the height of the peak is also associated with the g value, i.e., higher maxima occur for a lower SSA and higher g. This representation also evidences that when the particle size drops to a few tens of nanometers, the effect of the distance between them seems to overcome the effect of their number, i.e., the individual curves become overlapped.

**Figure 10:**
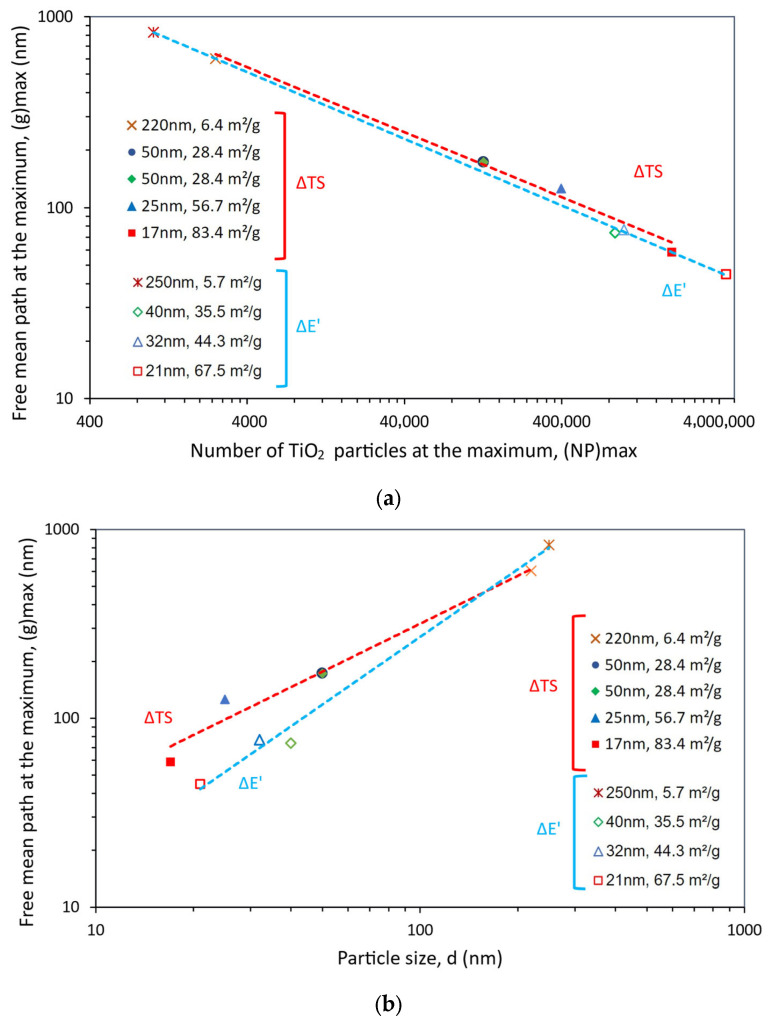
Power law relationship observed at the maximum property gain (ΔTS and ΔE’) for TiO_2_-filled epoxy composites, between (**a**) particle number, NP, and mean free path, g, and (**b**) particle diameter, d, and mean free path, g. To help the discussion, the data shown earlier in Figure 7 is included again.

The relationships between g and NP at the property maximum (i.e., g_max_ and NP_max_) and between g_max_ and the particle size, d, can be seen in Figure 10. To help the discussion, the data shown earlier in Figure 7 is included again. The expected negative power law relationship between NP_max_ and g_max_ (or the linear relationship between log NP_max_ and log g_max_), as well as the positive power law relationship between g_max_ and d at the property maximum, can also be seen for the storage modulus.

It can be seen in Figure 10a,b that the relationships between the interparticle distance at the property maxima (g_max_) and the particle size (d), as well as between g_max_ and NP_max_, are, for all practical purposes, the same for ΔTS and ΔE’, supporting the inferred presence of an “affected” matrix volume surrounding each particle, which depends on the particle size and increases with it. However, because the dependence of the stiffness E’ on the number of particles was found to be contrary to that of the strength, TS, it appears that, as g decreases (more particles in the polymeric matrix), the polymeric chains in these narrow gaps likely become more oriented and it would be easier for them to slip past each other (easier movement). This apparently results in a more flexible composite (a decrease in E’) but one with increased strength, TS (and maximum strain).

#### 4.2.2. Glass Transition Temperature

**Figure 11:**
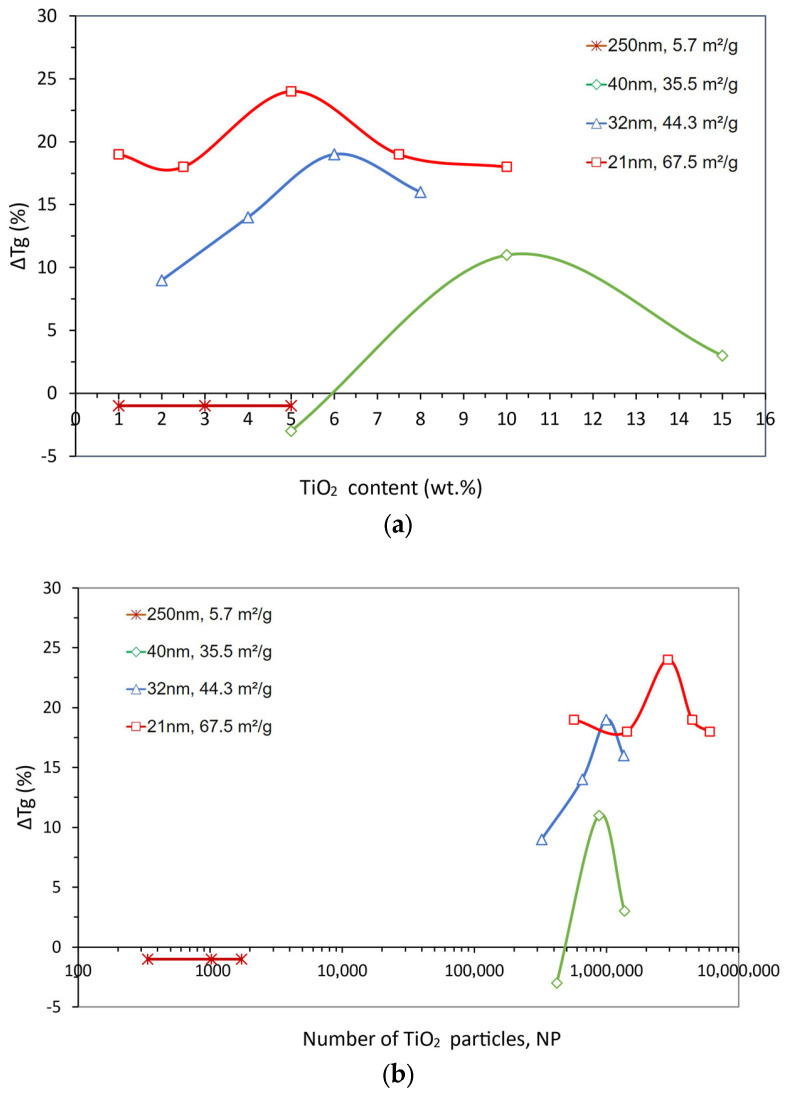
Glass transition temperature variation (ΔT_g_) for TiO_2_-filled epoxy composites as a function of (**a**) TiO_2_ weight content and (**b**) number of TiO_2_ particles for the particle size and SSA values shown.

To further clarify the influence of TiO_2_-particle downsizing on the structure–property relationships in TiO_2_-filled epoxy composites, reported data (selected from Table 5) for the glass transition temperature (T_g_) was similarly explored. Figure 11a,b depicts the T_g_ variation as a function of the TiO_2_ content and the number of particles, respectively.

Figure 11a shows that T_g_ seems to improve for the high SSA (i.e., for particles with smaller sizes), as seen earlier for the tensile strength (Figure 1 and Figure 4) but is contrary to the behavior of the storage modulus seen in Figure 8a. However, the curves appear to be flatter, and more so for the higher SSA, i.e., less sensitivity to the particles’ weight content.

From the perspective of the number of particles, shown in Figure 11b, the effect of lower thermal stability for the nanocomposites with bigger particles becomes even more evident. The difference in the size of particles with 44.3 and 35.5 m^2^/g SSA (32 and 40 nm, respectively) appears to be not significant enough to change their number for the highest property improvement despite the different weight contents that were used.

This analysis would suggest that the mechanisms for property enhancement of epoxy through the dispersion of nanoparticles are different for storage modulus and glass transition temperature. Thus, it is possible that the increase in T_g_ may be related to a reduction in the mean free path among particles, hence being favored by smaller particles that can stand closer to each other, thus promoting the loss in the flexibility of polymer chains that seems to favor T_g_.

## 5. Conclusions and Outlook

Technological advances have allowed the development of new nanoparticles and the improvement of processing techniques for the preparation and design of nanocomposites, and despite the fact that nanocomposites have been studied for decades, several authors have recently devoted their work to providing new findings in various fields, such as ballistics [146], sensors [147], water treatment [148], conducting polymer composites [149], synergetic effects of self/induced crystallization and nanoparticles on the mechanical properties of nanocomposites [150], mechanical properties increment based on carbon nanoparticles, nanosilicon, and cobalt [111,151,152,153,154], besides thorough, updated, state-of-the-art reviews [155,156].

The chronology of the articles reviewed from the literature clearly depicts this trend, as added nanoparticles with progressively smaller sizes are being investigated, and the consequent exponential gains in the properties of polymeric matrix composites are being reported. This work analyzed the particular case of titania nanoparticles added to epoxy matrices, seeking a better understanding of the observed improvement in important thermomechanical properties, namely the tensile stress (TS), the storage (elastic) modulus (E’) and the glass transition temperature (T_g_).

The reported composite property improvement due to the simple decrease in particle size, the so-called “scaling effect”, is attributed to surface energy effects, as the specific surface area of a given weight content of particles of the same material directly increases as their size decreases. What appears to have been overlooked so far is that the resulting number of smaller particles increases much faster, and what might have seemed a tiny weight of added particles is, in fact, a huge particle number and a very crowded system whose processing difficulties, often directly linked to a good dispersibility, might be tremendous, upsetting the delicate balance between best performance and economic viability.

It should be remembered that real particles may have a variety of sizes (represented by a flatter particle size distribution curve) and may agglomerate before or after addition to the polymeric matrices, all of which hinder homogeneous dispersions. These processing steps are very challenging, even with current technology. It would, therefore, not be hard to accept that the experimental results reported in the literature could be impaired by heterogeneous dispersions and/or agglomerations of uneven particles, resulting in composites that are prone to premature failure.

The significance of considering tiny amounts of smaller and well-dispersed particles within the polymer matrix was highlighted in this work in terms of the number of particles, NP, needed to reach the highest property gain for a given particle size, d (or specific surface area) and the likely mean free path (distance), g, between the closest particles, i.e., particle crowding. For all practical purposes, the same positive power law relationships between g and NP, as well as between g and d, at the property maximum were identified both for TS and E’, suggesting that matrix texturing around the particles increases with their size. However, the dependence of the stiffness on the number of particles was found to be contrary to that of the strength, suggesting that, as g decreases (more particles in the polymeric matrix), the likely forced orientation of polymeric chains apparently prompts easier slipping, resulting in a more flexible composite—but one with increased strength (and maximum strain). Surprisingly, the glass transition temperature appears to be less sensitive to particle weight content or crowding, being simply favored by smaller particles that can stand closer to each other, hence promoting the loss in the flexibility of polymer chains that favor the increase in T_g_.

Nowadays, given the varied techniques and materials used by each author, normalizing the results through particle crowding (the number of particles and distance among them) brings evidence that the particle size and particle content should be downscaled together and has an interesting potential to better compare the laboratory results and further the knowledge on such important processing–structure–property relationships.

## Figures and Tables

**Table 1:** Summary on the effect of particle dispersion method on the glass transition temperature (T_g_) of epoxy nanocomposites relative to that of the matrix.

Dispersion Method	Nanoparticles			ΔT_g_ (%)	Composites Preparation Remarks	Reference
	Type	Size (nm)	Content (wt.%)			
**Physical:** Melt-blending processing	SiC	<100	10	6	Prepared at 80 °C by 60 min stirring and 30 min sonication. T_g_: DMA (1 Hz, 35–250 °C, 5 °C/min).	[62]
	CaCO_3_	40–70	6	0	Prepared at 80 °C by 60 min stirring and sonication. T_g_: DMA (1 Hz, 30–300 °C, 5 °C/min).	[61]
	SiO_2_	100	5	5	Prepared at an elevated temperature by 30 min sonication. T_g_: DSC (25–200 °C, 5 °C/min).	[59]
	CB ^a^	30	2	2	Prepared at an elevated temperature by 5 min sonication. T_g_: DSC (25–200 °C, 5 °C/min).	[59]
**Physical:** Mechanical-aided solvent processing	ZrO_2_	45	6	11	Prepared in butanone with 10 min of stirring and 60 min sonication. T_g_: DMA (1 Hz, 28–100 °C, 2 °C/min).	[82]
	Al_2_O_3_	80	1	21	Prepared in liquid resin by high-speed mixing. T_g_: DMA (1 Hz, 40–130 °C, 2 °C/min).	[77]
	SiO_2_	12	6	0	Prepared in acetone by mechanical mixing and sonication. T_g_: DMA (1 Hz, 20–175 °C).	[76]
	TiO_2_	<10	3	21	Prepared in benzyl alcohol by 5 min high-speed mixing and 5 min sonication. T_g_: DMTA.	[74]
**Chemical:** Covalent bonding through particle functionalization	SiO_2_	7	7	15	Prepared by covalent bonding between functionalized SiO_2_ and resin by 10 min mixing and 30 min sonication. T_g_: DMA (1 Hz, 25–200 °C, 5 °C/min).	[89]
	GO	n/a	0.5	5	Prepared by covalent bonding between functionalized GO and resin by 30 min mixing. T_g_: DMA.	[90]
	TiO_2_	25	5	43	Prepared by covalent bonding between functionalized TiO_2_ and resin by 15 min stirring. T_g_: DSC (N_2_ atmosphere, 20–200 °C, 10 °C/min).	[22]
	CNT ^b^	*d*: 30 nm L: 15 µm	0.5	34	Prepared by covalent bonding between functionalized CNT and resin by 60 min stirring. T_g_: DSC.	[88]
**Chemical:** In situ matrix formation in a particle suspension	CNT	*d*: 9.5 nm L: 1.5 µm	0.2	27	Prepared by in situ polymer formation in a CNT suspension. T_g_: DMTA (1 Hz, 25–200 °C, 2 °C/min).	[95]
	GO	n/a	0.5	40	Prepared by in situ polymer formation in a GO suspension. T_g_: DMA (1 Hz, 5 °C/min).	[96]
	TiO_2_	<25	7.6	25	Prepared by in situ polymer formation in a TiO_2_ suspension. T_g_: DMTA (1 Hz, 5 °C/min).	[97]
	rGO ^c^	n/a	1	0	Prepared by in situ polymer formation in a rGO suspension. T_g_: DSC.	[92]

^a^ CB: carbon black; ^b^ for carbon nanotubes, *d* is the diameter, and L is the length; ^c^ rGO: reduced graphene oxide.

**Table 2:** Comparison of relative changes in tensile strength (ΔTS) for epoxy matrix nanocomposites as a function of type, size and content of nanoparticles.

Type	Size (nm)	Content (wt.%)	ΔTS (%)	Reference
**T_i_O_2_**	220	1; 3; 5; 10	2; 4; 13; 9	[100]
	190	2; 4; 6; 8; 10	45; 62; 31; 14; 7	[100]
	50	1; 3; 5; 10	7; 14; 11; 9	[100]
	50	1; 3; 5	5; 15; 9	[101]
	25	0.5; 1; 3; 5	11; 18; 15; 6	[102]
	17	1; 3; 5; 10	13; 14; 12; 6	[100]
	15	1; 2; 3; 4; 5; 6	51; 71; 19; 11; 7; 21	[103]
**S_i_O_2_**	30	0.5; 1; 2; 3	4; 9; 11; 0	[104]
	17	0.5; 0.75; 1; 3	12; 42; −6; −14	[105]
	15	2; 4; 6; 8	14; 27; 10; 6	[82]
	15	1; 2; 3; 4; 5	9; 13; 117; −14; 14	[106]
	15	2; 4; 6; 8	15; 29; 3; −12	[107]
	15	0.5; 1; 1.5; 3; 5	1; 24; 26; 31; 27	[108]
GO *	500 T: 1.2 nm	0.1; 0.2; 0.5; 1	7; 9; 11; 15	[109]
	500 T: 1.2 nm	0.1; 0.2; 0.5; 1	3; 4; 7; −6	[109]
	1.5 × 10^3^ T: 2.8 nm	0.025; 0.05; 0.1; 0.2	2; 10; 19; 14	[110]
	1.5 × 10^3^ T: 3.5 nm	0.025; 0.05; 0.1; 0.2	20; 29; 17; 5	[110]
	3.0 × 10^3^ T: 1.2 nm	0.1; 0.3; 0.5; 0.7; 1	3; 2; 12; 6; 4; 2	[111]
	5.0 × 10^3^ T: 4.0 nm	0.1; 0.25; 0.5; 1	11; 11; 13; −1;5	[112]
GNP *	5.0 × 10^3^ T: 25 nm	0.25; 0.5; 1; 1.5	20; 11; 1; −6	[113]
	5.0 × 10^3^ T: 25 nm	0.1; 0.25; 0.5; 1	5; 13; 10; 2; 7	[114]
	5.0 × 10^3^ T: 25 nm	0.1; 0.25; 0.5; 1	−1; −13; −8; 0	[115]
	5.0 × 10^3^ T: 25 nm	0.1; 0.25; 0.5; 1	0; 20; 11; 1	[115]

* For GO and GNP; T is the sheet thickness.

**Table 3:** Comparison of relative changes in storage modulus (E’), tan δ and glass transition temperature (T_g_) for epoxy matrix nanocomposites as a function of type, size and content of nanoparticles.

Type			Properties (%)			Reference
	Size (nm)	Content (wt.%)	ΔE’	Δtan δ	ΔT_g_	
TiO_2_	250	1; 3; 5	20; 52; 40	Unchanged	−1	[116]
	21	1; 2.5; 5; 7.5; 10	−15; 4; 8; 12; −35	110; 80; 60; 70; 30	19; 18; 24; 19; 18	[114]
	30–40	5; 10; 15	18; 30; −10	−7; 7; −9	−3; 11; 3	[117]
	32	2; 4; 6; 8	9; 21; 22; 15	−6; −5; −9; −5	9; 14; 19; 16	[118]
	17	0.2; 0.4; 0.6	25; 31; 45	6; −4; −3	−3; 2; 3	[13]
	30	0.1; 0.5; 1; 1.5; 2; 2.5; 3; 3.5; 4	not reported	215; 115; 138; 246; 123; 246; 85; 77; 119	−11; −5; −7; −7	[117,119]
SiO_2_	90	1; 3; 5; 7	8; 23; 15; 15	2; −9; −2; 12	1; 2; 0; −8	[120]
	40	1; 3; 5	8; 14; 44	30; 43; 22	32; 36; 41	[121]
	70	0.5; 2; 5	not reported	−14; −27; −28	7; 16; 16	[122]
	40	1; 2; 3; 4; 5; 6; 7; 8	5; 8; 14; 14; 17; 11; 1; −20	−10; −9; 0; 3; 0; −9; −17; −19	7; 10; 10; 12; 0; 7; 0; 1	[85]
	15	1; 2; 4	not reported	−15; −16; −21	3; 3; 3	[123]
GO *	1.3 × 10^3^ T: 1 nm	0.1; 0.5; 1; 1.5	30; 35; 35; 39	10; −7; −11; −21	−1; −1; −3; −11	[124]
	W: 5 µm L: 25 µm T: 1.7 nm	0.05; 0.1; 0.2; 0.4	6; 6; 56; 44	13; 20; 22; 9	1; 0; 13; 5	[125]
	20 × 10^3^ T: 9 nm	0.1; 0.25; 0.5	not reported	−15; −15; −11	5; 6; 5	[126]
	20 × 10^3^ T: 11 nm	0.1; 0.25; 0.5	not reported	−16; −11; −22	6; 5; 7	[126]
	2.5 × 10^3^ T: 12.3 nm	0.4; 0.8; 1.2	25; 81; 53	−7; −16; −7	5; 9; 6	[127]
	12 × 10^3^ T: 12 nm	0.1; 0.3; 0.5	54; 48; 46	100; 77; 110	18; 15; 3	[128]
GNP *	5 × 10^3^ T: 10 nm	1; 3; 5	2; 7; 16	−2; −7; −10	1; 1; 2	[129]
	4.5 × 10^3^ T: 12 µm	0.1; 0.3; 0.5; 1	24; 30; 18; 12	−7; −12; −14; −12	4; 6; 5; 4	[130]
	25 × 10^3^ T: 6–8 nm	0.1; 0.25; 0.5; 1	not reported	5; −9; −35; −25	1; 4; 7; 1	[131]
	4.5 × 10^3^ T: 12 nm	0.025; 0.1; 0.2	6; 18; 13	−26; −50; −38	6; 19; 15	[132]
	5 × 10^3^ T: 6 nm	0.1; 0.3; 0.5; 0.7	8; 10; 25; −9	−5; −9; −18; −15	2; 4; 5; 3	[133]

* Is for GO and GNP; T is the sheet thickness; W is the width; L is the length.

**Table 4:** Comparison of relative changes in tensile strength (ΔTS) for TiO_2_-epoxy nanocomposites as a function of particle size and added particle content (particle number (NP), mean free path (g) and specific surface area (SSA) were calculated).

Size (nm)	Content (wt.%)	ΔTS (%)	NP	g (nm)	SSA (m^2^/g)	Reference
220	1	2	4.95 × 10^2^	1159	6.4	[100]
	3	4	2.34 × 10^3^	731		
	5	13	3.96 × 10^3^	578		
	10	9	4.52 × 10^5^	406		
50	1	7	4.95 × 10^2^	263	28.4	[100]
	3	14	2.34 × 10^3^	166		
	5	11	3.96 × 10^3^	131		
	10	9	4.52 × 10^5^	92		
50	1	5	1.07 × 10^6^	263	28.4	[101]
	3	15	4.76 × 10^6^	166		
	5	9	8.05 × 10^6^	131		
25	0.5	11	7.79 × 10^5^	173	56.7	[102]
	1	18	1.56 × 10^6^	132		
	3	15	4.76 × 10^6^	83		
	5	6	8.05 × 10^6^	66		
17	1	13	4.95 × 10^2^	90	83.4	[100]
	3	14	1.51 × 10^3^	57		
	5	12	3.96 × 10^3^	45		
	10	6	4.52 × 10^5^	31		

**Table 5:** Comparison of relative change in storage modulus (ΔE’) and glass transition temperature (ΔT_g_) for TiO_2_-epoxy nanocomposites as a function of particle size and added particle content (particle number (NP), mean free path (g) and specific surface area (SSA) were calculated).

Size (nm)	ΔE’ (%)	ΔT_g_ (%)	Content (wt.%)	NP	g (nm)	SSA (m^2^/g)	Reference
250	20	−1	1	3.38 × 10^2^	1317		[116]
	52	−1	3	1.03 × 10^3^	831	5.7	
	40	−1	5	1.74 × 10^3^	657		
40	18	−3	5	4.25 × 10^5^	105		[117]
	30	11	10	8.82 × 10^5^	74	35.5	
	−10	3	15	1.38 × 10^6^	58		
32	9	9	2	3.24 × 10^5^	127	44.3	[118]
	21	14	4	6.58 × 10^5^	93		
	22	19	6	1.00 × 10^6^	77		
	15	16	8	1.36 × 10^6^	67		
21	−15	19	1	5.70 × 10^5^	111		[114]
	4	18	2.5	1.44 × 10^6^	76		
	8	24	5	2.93 × 10^6^	55	67.5	
	12	19	7.5	4.49 × 10^6^	45		
	−35	18	10	6.10 × 10^6^	39		

## Data Availability

The data are contained within the article.

## References

[1] Lobo R.F.M. (2009). Nanotechnology and Nanophysics. Concepts of Modern Nanoscience.

[2] Al-Rodhan N.R.F. (2011). Materials Science. The Politics of Emerging Strategic Technologies.

[3] Lau K., Hung P., Zhu M.-H., Hui D. (2018). Properties of Natural Fibre Composites for Structural Engineering Applications. Compos. Part B Eng..

[4] Yun M., Sas H., Simacek P., Advani S.G. (2017). Characterization of 3D Fabric Permeability with Skew Terms. Compos. Part A Appl. Sci. Manuf..

[5] Kuilla T., Bhadra S., Yao D., Kim N.H., Bose S., Lee J.H. (2010). Recent Advances in Graphene Based Polymer Composites. Prog. Polym. Sci..

[6] Saba N., Jawaid M., Alothman O.Y., Paridah M.T. (2016). A Review on Dynamic Mechanical Properties of Natural Fibre Reinforced Polymer Composites. Constr. Build. Mater..

[7] Ibeh C.C., Bubacz M. (2008). Current Trends in Nanocomposite Foams. J. Cell. Plast..

[8] Abdelkarim M.F., Nasrat L.S., Elkhodary S.M., Soliman A.M., Hassan A.M., Mansour S.H. (2015). Volume Resistivity and Mechanical Behavior of Epoxy Nanocomposite Materials. Eng. Technol. Appl. Sci. Res..

[9] Rafique I., Kausar A., Muhammad B. (2016). Epoxy Resin Composite Reinforced with Carbon Fiber and Inorganic Filler: Overview on Preparation and Properties. Polym.-Plast. Technol. Eng..

[10] Masala O., Seshadri R. (2004). Synthesis Routes for Large Volumes of Nanoparticles. Annu. Rev. Mater. Res..

[11] Pinto D., Bernardo L., Amaro A., Lopes S. (2015). Mechanical Properties of Epoxy Nanocomposites Using Titanium Dioxide as Reinforcement—A Review. Constr. Build. Mater..

[12] Goud K.Y., Reddy K.K., Satyanarayana M., Kummari S., Gobi K.V. (2020). A Review on Recent Developments in Optical and Electrochemical Aptamer-Based Assays for Mycotoxins Using Advanced Nanomaterials. Microchim. Acta.

[13] Prasad V., Sekar K., Varghese S., Joseph M.A. (2020). Evaluation of Interlaminar Fracture Toughness and Dynamic Mechanical Properties of Nano TiO_2_ Coated Flax Fibre Epoxy Composites. Polym. Test..

[14] Hunain M.B., Abass B.A., Akhudair J.M. (2021). Experimental and Numerical Studies of Fatigue Properties of Carbon/Glass Fiber/Epoxy Hybrid Composites Enhanced with Nano TiO_2_ powder. Diagnostyka.

[15] Al-Zubaydi A.S.J., Salih R.M., Al-Dabbagh B.M. (2021). Effect of Nano TiO_2_ Particles on the Properties of Carbon Fiber-Epoxy Composites. Prog. Rubber Plast. Recycl. Technol..

[16] Akhter F., Rao A.A., Abbasi M.N., Wahocho S.A., Mallah M.A., Anees-ur-Rehman H., Chandio Z.A. (2022). A Comprehensive Review of Synthesis, Applications and Future Prospects for Silica Nanoparticles (SNPs). Silicon.

[17] Aziz I., Duran H., Saleem M., Yameen B., Arshad S.N. (2021). The Role of Interface on Dynamic Mechanical Properties, Dielectric Performance, Conductivity, and Thermal Stability of Electrospun Carbon Nanofibers Reinforced Epoxy. Polym. Compos..

[18] Zhang X.R., Pei X.Q., Wang Q.H. (2009). Friction and Wear Studies of Polyimide Composites Filled with Short Carbon Fibers and Graphite and Micro SiO_2_. Mater. Des..

[19] Fernández-Álvarez M., Velasco F., Bautista A. (2021). Performance of Ultraviolet Exposed Epoxy Powder Coatings Functionalized with Silica by Hot Mixing. J. Mater. Res. Technol..

[20] Xie Z., Li H., Zhang S., Gong A., Liu P., Peng Z., Wang Q. Research on Electrical Properties of Surface-Modified Nano-SiO_2_/Epoxy Composites. Proceedings of the 7th IEEE International Conference on High Voltage Engineering and Application, ICHVE 2020.

[21] Zhou S., Wu L., Xiong M., He Q., Chen G. (2005). Dispersion and UV-VIS Properties of Nanoparticles in Coatings. J. Dispers. Sci. Technol..

[22] Wang T., Liu C., Li D., Hou Y., Zhang G., Zhang B. (2020). Nano ZnO/Epoxy Coating to Promote Surface Charge Dissipation on Insulators in DC Gas-Insulated Systems. IEEE Trans. Dielectr. Electr. Insul..

[23] Ammar S., Ramesh K., Vengadaesvaran B., Ramesh S., Arof A.K. (2016). Amelioration of Anticorrosion and Hydrophobic Properties of Epoxy/PDMS Composite Coatings Containing Nano ZnO Particles. Prog. Org. Coat..

[24] Theerthagiri J., Salla S., Senthil R.A., Nithyadharseni P., Madankumar A., Arunachalam P., Maiyalagan T., Kim H.-S. (2019). A Review on ZnO Nanostructured Materials: Energy, Environmental and Biological Applications. Nanotechnology.

[25] Babaei I., Madanipour M., Farsi M., Farajpoor A. (2014). Physical and Mechanical Properties of Foamed HDPE/Wheat Straw Flour/Nanoclay Hybrid Composite. Compos. Part B Eng..

[26] Klingshirn C.F. (2007). ZnO: Material, Physics and Applications. ChemPhysChem.

[27] Battez A.H., González R., Viesca J.L., Fernández J.E., Fernández J.M.D., Machado A., Chou R., Riba J. (2008). CuO, ZrO_2_ and ZnO Nanoparticles as Antiwear Additive in Oil Lubricants. Wear.

[28] Cangul I.T., Gul N.Y., Topal A., Yilmaz R. (2006). Evaluation of the Effects of Topical Tripeptide-Copper Complex and Zinc Oxide on Open-Wound Healing in Rabbits. Vet. Dermatol..

[29] De Liedekerke M., Auer G., Woditsch P., Westerhaus A., Kischkewitz J., Griebler W.-D. (2006). Pigments, Inorganic, 2. White Pigments. Ullmann’s Encycl. Ind. Chem..

[30] Zhang Z., Yang X., Fu Y., Du K. (2015). Ultrathin Molybdenum Diselenide Nanosheets Anchored on Multi-Walled Carbon Nanotubes as Anode Composites for High Performance Sodium-Ion Batteries. J. Power Sources.

[31] Jahanmardi R., Kangarlou B., Dibazar A.R. (2013). Effects of Organically Modified Nanoclay on Cellular Morphology, Tensile Properties, and Dimensional Stability of Flexible Polyurethane Foams. J. Nanostruct. Chem..

[32] Hossain Z., Zaman M., Hawa T., Saha M.C. (2014). Evaluation of Moisture Susceptibility of Nanoclay-Modified Asphalt Binders through the Surface Science Approach. J. Mater. Civ. Eng..

[33] LibreTexts^TM^. https://chem.libretexts.org/Bookshelves/General_Chemistry/Chemistry_1e_%28OpenSTAX%29/10%3A_Liquids_and_Solids/10.6%3A_Lattice_Structures_in_Crystalline_Solids.

[34] Najafi N., Heuzey M.C., Carreau P.J. (2012). Polylactide (PLA)-Clay Nanocomposites Prepared by Melt Compounding in the Presence of a Chain Extender. Compos. Sci. Technol..

[35] Vasiliu S., Racovita S., Gugoasa I.A., Lungan M.A., Popa M., Desbrieres J. (2021). The Benefits of Smart Nanoparticles in Dental Applications. Int. J. Mol. Sci..

[36] Tipa C., Cidade M.T., Vieira T., Silva J.C., Soares P.I.P., Borges J.P. (2021). A New Long-Term Composite Drug Delivery System Based on Thermo-Responsive Hydrogel and Nanoclay. Nanomaterials.

[37] Allen M.J., Tung V.C., Kaner R.B. (2010). Honeycomb Carbon: A Review of Graphene. Chem. Rev..

[38] Kumar A., Sharma K., Dixit A.R. (2019). A Review of the Mechanical and Thermal Properties of Graphene and Its Hybrid Polymer Nanocomposites for Structural Applications. J. Mater. Sci..

[39] Kim J., Kim J., Song S., Zhang S., Cha J., Kim K., Yoon H., Jung Y., Paik K.W., Jeon S. (2017). Strength Dependence of Epoxy Composites on the Average Filler Size of Non-Oxidized Graphene Flake. Carbon.

[40] Subha S., Singh D., Venkatanarayanan P.S. (2018). Thermal, Ablation and Mechanical Properties of Carbon-Phenolic Composites Reinforced with Zirconia Coated Graphene Nanoplatelets. Mater. Res. Express.

[41] Jang B.Z., Zhamu A. (2008). Processing of Nanographene Platelets (NGPs) and NGP Nanocomposites: A Review. J. Mater. Sci..

[42] Goenka S., Sant V., Sant S. (2014). Graphene-Based Nanomaterials for Drug Delivery and Tissue Engineering. J. Control. Release.

[43] Moradi S.E. (2015). Highly Efficient Removal of Amoxicillin from Water by Magnetic Graphene Oxide Adsorbent. Chem. Bull. ‘Politeh. Timis. ROMANIA Ser. Chem. Environ. Eng..

[44] Akhavan O., Ghaderi E., Aghayee S., Fereydooni Y., Talebi A. (2012). The Use of a Glucose-Reduced Graphene Oxide Suspension for Photothermal Cancer Therapy. J. Mater. Chem..

[45] Ha H., Ellison C.J. (2018). Polymer/Graphene Oxide (GO) Thermoset Composites with GO as a Crosslinker. Korean J. Chem. Eng..

[46] Papageorgiou D.G., Li Z., Liu M., Kinloch I.A., Young R.J. (2020). Mechanisms of Mechanical Reinforcement by Graphene and Carbon Nanotubes in Polymer Nanocomposites. Nanoscale.

[47] Bhattacharya M. (2016). Polymer Nanocomposites-A Comparison between Carbon Nanotubes, Graphene, and Clay as Nanofillers. Materials.

[48] Zurutuza A., Marinelli C. (2014). Challenges and Opportunities in Graphene Commercialization. Nat. Nanotechnol..

[49] Boaretto J., Fotouhi M., Tende E., Aver G.F., Marcon V.R.R., Cordeiro G.L., Bergmann C.P., de Camargo F.V. (2021). Biomimetics and Composite Materials toward Efficient Mobility: A Review. J. Compos. Sci..

[50] Qiu P., Mao C. (2010). Biomimetic Branched Hollow Fibers Templated by Self-Assembled Fibrous Polyvinylpyrrolidone Structures in Aqueous Solution. ACS Nano.

[51] Dikshit V., Bhudolia S.K., Joshi S.C. (2017). Multiscale Polymer Composites: A Review of the Interlaminar Fracture Toughness Improvement. Fibers.

[52] Zhu Y., Bakis C.E., Adair J.H. (2012). Effects of Carbon Nanofiller Functionalization and Distribution on Interlaminar Fracture Toughness of Multi-Scale Reinforced Polymer Composites. Carbon.

[53] Ferreira F.V., Dufresne A., Pinheiro I.F., Souza D.H.S., Gouveia R.F., Mei L.H.I., Lona L.M.F. (2018). How Do Cellulose Nanocrystals Affect the Overall Properties of Biodegradable Polymer Nanocomposites: A Comprehensive Review. Eur. Polym. J..

[54] Azani N.F.S.M., Hussin M.H. (2021). Comparison of Cellulose Nanocrystal (CNC) Filler on Chemical, Mechanical, and Corrosion Properties of Epoxy-Zn Protective Coatings for Mild Steel in 3.5% NaCl Solution. Cellulose.

[55] Mousavi S.R., Nejad S.F., Jafari M., Paydayesh A. (2021). Polypropylene/Ethylene Propylene Diene Monomer/Cellulose Nanocrystal Ternary Blend Nanocomposites: Effects of Different Parameters on Mechanical, Rheological, and Thermal Properties. Polym. Compos..

[56] Li Y., Gong Q., Liu X., Xia Z., Yang Y., Chen C., Qian C. (2021). Wide Temperature-Tolerant Polyaniline/Cellulose/Polyacrylamide Hydrogels for High-Performance Supercapacitors and Motion Sensors. Carbohydr. Polym..

[57] Cheng R., Zeng J., Wang B., Li J., Cheng Z., Xu J., Gao W., Chen K. (2021). Ultralight, Flexible and Conductive Silver Nanowire/Nanofibrillated Cellulose Aerogel for Multifunctional Strain Sensor. Chem. Eng. J..

[58] Ferreira F.V., Lona L.M.F., Pinheiro I.F., de Souza S.F., Mei L.H.I. (2019). Polymer Composites Reinforced with Natural Fibers and Nanocellulose in the Automotive Industry: A Short Review. J. Compos. Sci..

[59] Sun Y., Zhang Z., Moon K.S., Wong C.P. (2004). Glass Transition and Relaxation Behavior of Epoxy Nanocomposites. J. Polym. Sci. B Polym. Phys..

[60] Jordan J., Jacob K.I., Tannenbaum R., Sharaf M.A., Jasiuk I. (2005). Experimental Trends in Polymer Nanocomposites—A Review. Mater. Sci. Eng. A.

[61] Jin F.-L., Park S.-J., Paik S.-J. (2009). Thermal Stability of Trifunctional Epoxy Resins Modified with Nanosized Calcium Carbonate. Bull. Korean Chem. Soc.

[62] Jin H., Mangun C.L., Stradley D.S., Moore J.S., Sottos N.R., White S.R. (2012). Self-Healing Thermoset Using Encapsulated Epoxy-Amine Healing Chemistry. Polymer.

[63] Jouni M., Djurado D., Massardier V., Boiteux G. (2017). A Representative and Comprehensive Review of the Electrical and Thermal Properties of Polymer Composites with Carbon Nanotube and Other Nanoparticle Fillers. Polym. Int..

[64] Restrepo I., Benito N., Medinam C., Mangalaraja R.V., Flores P., Rodriguez-Llamazares S. (2017). Development and Characterization of Polyvinyl Alcohol Stabilized Polylactic Acid/ZnO Nanocomposites. Mater. Res. Express.

[65] Jiang S., Chen Y., Duan G., Mei C., Greiner A., Agarwal S. (2018). Electrospun Nanofiber Reinforced Composites: A Review. Polym. Chem..

[66] Zhou Y., Lei L., Yang B., Li J., Ren J. (2018). Preparation and Characterization of Polylactic Acid (PLA) Carbon Nanotube Nanocomposites. Polym. Test..

[67] Blattmann C.O., Pratsinis S.E. (2019). Nanoparticle Filler Content and Shape in Polymer Nanocomposites. KONA Powder Part. J..

[68] Fu S., Sun Z., Huang P., Li Y., Hu N. (2019). Some Basic Aspects of Polymer Nanocomposites: A Critical Review. Nano Mater. Sci..

[69] Lepcio P., Ondreáš F., Zárybnická K., Zbončák M., Svatík J., Jančář J. (2021). Phase Diagram of Bare Particles in Polymer Nanocomposites: Uniting Solution and Melt Blending. Polymer.

[70] Sharip N.S., Ariffin H., Yasim-Anuar T.A.T., Andou Y., Shirosaki Y., Jawaid M., Tahir P.M., Ibrahim N.A. (2021). Melt-vs. Non-Melt Blending of Complexly Processable Ultra-High Molecular Weight Polyethylene/Cellulose Nanofiber Bionanocomposite. Polymer.

[71] Wichmann M.H.G., Sumfleth J., Gojny F.H., Quaresimin M., Fiedler B., Schulte K. (2006). Glass-Fibre-Reinforced Composites with Enhanced Mechanical and Electrical Properties–Benefits and Limitations of a Nanoparticle Modified Matrix. Eng. Fract. Mech..

[72] Bittmann B., Haupert F., Schlarb A.K. (2009). Ultrasonic Dispersion of Inorganic Nanoparticles in Epoxy Resin. Ultrason Sonochem.

[73] Qian H., Greenhalgh E.S., Shaffer M.S.P., Bismarck A. (2010). Carbon Nanotube-Based Hierarchical Composites: A Review. J. Mater. Chem..

[74] Morselli D., Bondioli F., Fiorini M., Messori M. (2012). Poly(Methyl Methacrylate)-TiO_2_ Nanocomposites Obtained by Non-Hydrolytic Sol-Gel Synthesis: The Innovative Tert-Butyl Alcohol Route. J. Mater. Sci..

[75] Tang Y., Ye L., Zhang Z., Friedrich K. (2013). Interlaminar Fracture Toughness and CAI Strength of Fibre-Reinforced Composites with Nanoparticles—A Review. Compos. Sci. Technol..

[76] Zamanian M., Mortezaei M., Salehnia B., Jam J.E. (2013). Fracture Toughness of Epoxy Polymer Modified with Nanosilica Particles: Particle Size Effect. Eng. Fract. Mech..

[77] Liang M., Wong K.L. (2017). Study of Mechanical and Thermal Performances of Epoxy Resin Filled with Micro Particles and Nanoparticles. Energy Procedia.

[78] Mittal G., Rhee K.Y., Mišković-Stanković V., Hui D. (2018). Reinforcements in Multi-Scale Polymer Composites: Processing, Properties, and Applications. Compos. B Eng..

[79] Harito C., Bavykin D.V., Yuliarto B., Dipojono H.K., Walsh F.C. (2019). Polymer Nanocomposites Having a High Filler Content: Synthesis, Structures, Properties, and Applications. Nanoscale.

[80] Liu K., Macosko C.W. (2019). Can Nanoparticle Toughen Fiber-Reinforced Thermosetting Polymers?. J. Mater. Sci..

[81] Di Boon Y., Joshi S.C. (2020). A Review of Methods for Improving Interlaminar Interfaces and Fracture Toughness of Laminated Composites. Mater. Today Commun..

[82] Singh S.K., Kumar A., Jain A. (2021). Mechanical and Viscoelastic Properties of SiO_2_/Epoxy Nanocomposites Post-Cured at Different Temperatures. Plast. Rubber Compos..

[83] Lim J.V., Bee S.T., Sin L.T., Ratnam C.T., Hamid Z.A.A. (2021). A Review on the Synthesis, Properties, and Utilities of Functionalized Carbon Nanoparticles for Polymer Nanocomposites. Polymers.

[84] da Luz F.S., da Costa Garcia Filho F., del-Río M.T.G., Nascimento L.F.C., Pinheiro W.A., Monteiro S.N. (2020). Graphene-Incorporated Natural Fiber Polymer Composites: A First Overview. Polymers.

[85] Rong M.Z., Zhang M.Q., Ruan W.H. (2006). Surface Modification of Nanoscale Fillers for Improving Properties of Polymer Nanocomposites: A Review. Mater. Sci. Technol..

[86] Sprenger S. (2013). Epoxy Resin Composites with Surface-Modified Silicon Dioxide Nanoparticles: A Review. J Appl. Polym. Sci..

[87] Kango S., Kalia S., Celli A., Njuguna J., Habibi Y., Kumar R. (2013). Surface Modification of Inorganic Nanoparticles for Development of Organic-Inorganic Nanocomposites—A Review. Prog. Polym. Sci..

[88] Roy K., Jatejarungwong C., Potiyaraj P. (2018). Development of Highly Reinforced Maleated Natural Rubber Nanocomposites Based on Sol–Gel-Derived Nano Alumina. J. Appl. Polym. Sci..

[89] Cheon J., Kim M. (2021). Impact Resistance and Interlaminar Shear Strength Enhancement of Carbon Fiber Reinforced Thermoplastic Composites by Introducing MWCNT-Anchored Carbon Fiber. Compos. B Eng..

[90] Liang X., Li X., Tang Y., Zhang X., Wei W., Liu X. (2022). Hyperbranched Epoxy Resin-Grafted Graphene Oxide for Efficient and All-Purpose Epoxy Resin Modification. J. Colloid Interface Sci..

[91] Meer S., Kausar A., Iqbal T. (2016). Attributes of Polymer and Silica Nanoparticle Composites: A Review. Polym.-Plast. Technol. Eng..

[92] Guo Y., Bao C., Song L., Yuan B., Hu Y. (2011). In Situ Polymerization of Graphene, Graphite Oxide, and Functionalized Graphite Oxide into Epoxy Resin and Comparison Study of on-the-Flame Behavior. Ind. Eng. Chem. Res..

[93] Roghani-Mamaqani H., Haddadi-Asl V., Salami-Kalajahi M. (2012). In Situ Controlled Radical Polymerization: A Review on Synthesis of Well-Defined Nanocomposites. Polym. Rev..

[94] Bhanvase B.A., Sonawane S.H. (2014). Ultrasound Assisted in Situ Emulsion Polymerization for Polymer Nanocomposite: A Review. Chem. Eng. Process. Process Intensif..

[95] Prolongo S.G., Díaz-Maroto C.G., Jiménez-Suárez A. (2021). Electroactive Shaping and Shape Memory of Sequential Dual-Cured off-Stoichiometric Epoxy/CNT Composites. J. Mater. Res. Technol..

[96] Romero-Zúñiga G.Y., Navarro-Rodríguez D., Treviño-Martínez M.E. (2022). Enhanced Mechanical Performance of a DGEBA Epoxy Resin-Based Shape Memory Polymer by Introducing Graphene Oxide via Covalent Linking. J. Appl. Polym. Sci..

[97] Morselli D., Bondioli F., Sangermano M., Roppolo I., Messori M. (2014). Epoxy Resins Reinforced with TiO_2_ Generated by Nonhydrolytic Sol-Gel Process. J. Appl. Polym. Sci..

[98] Szeluga U., Kumanek B., Trzebicka B. (2015). Synergy in Hybrid Polymer/Nanocarbon Composites. A Review. Compos. Part A Appl. Sci. Manuf..

[99] Kumar D., Babu G., Krishnan S. (2019). Study on Mechanical & Thermal Properties of PCL Blended Graphene Biocomposites. Polimeros.

[100] Al-Turaif H.A. (2010). Effect of Nano TiO_2_ Particle Size on Mechanical Properties of Cured Epoxy Resin. Prog. Org. Coat..

[101] Abass B.A., Hunain M.B., Khudair J.M.A. (2021). Effects of Titanium Dioxide Nanoparticles on the Mechanical Strength of Epoxy Hybrid Composite Materials Reinforced with Unidirectional Carbon and Glass Fibers. IOP Conf. Ser. Mater. Sci. Eng..

[102] Choi Y.M., Hwangbo S.A., Lee T.G., Ham Y.B. (2021). Effect of Particle Size on the Mechanical Properties of TiO_2_–Epoxy Nanocomposites. Materials.

[103] Pulikkottil E.J. (2019). The Effect of Nano TiO_2_ Filler Weight on the Mechanical Properties of Chopped Strand Mat Reinforced Epoxy Composite. Int. J. Res..

[104] Yildirim F., Aydin M., Avci A. (2017). Mechanical Properties of Nano-SiO_2_ Reinforced 3D Glass Fiber/Epoxy Composites. Int. J. Mater. Res..

[105] Lazar P.J.L., Sengottuvelu R., Natarajan E. (2018). Assessments of Secondary Reinforcement of Epoxy Matrix-Glass Fibre Composite Laminates through Nanosilica (SiO_2_). Materials.

[106] Zheng J., Zhang X., Cao J., Chen R., Aziz T., Fan H., Bittencourt C. (2021). Behavior of Epoxy Resin Filled with Nano-SiO_2_ Treated with a Eugenol Epoxy Silane. J. Appl. Polym. Sci..

[107] Singh S.K., Singh D., Kumar A., Jain A. (2019). An Analysis of Mechanical and Viscoelastic Behaviour of Nano-SiO_2_ Dispersed Epoxy Composites. Silicon.

[108] Suresha B., Divya G.S., Hemanth G., Somashekar H.M. (2021). Physico-Mechanical Properties of Nano Silica-Filled Epoxy-Based Mono and Hybrid Composites for Structural Applications. Silicon.

[109] Li W., Dichiara A., Bai J. (2013). Carbon Nanotube-Graphene Nanoplatelet Hybrids as High-Performance Multifunctional Reinforcements in Epoxy Composites. Compos. Sci. Technol..

[110] Yan L., Zhou Y., Zhang X., Zou H., Chen Y., Liang M. (2019). Effect of Graphene Oxide with Different Exfoliation Levels on the Mechanical Properties of Epoxy Nanocomposites. Polym. Bull..

[111] Eqra R., Moghim M.H., Eqra N. (2021). A Study on the Mechanical Properties of Graphene Oxide/Epoxy Nanocomposites. Polym. Polym. Compos..

[112] Bortz D.R., Heras E.G., Martin-Gullon I. (2012). Impressive Fatigue Life and Fracture Toughness Improvements in Graphene Oxide/Epoxy Composites. Macromolecules.

[113] Shen M.Y., Chang T.Y., Hsieh T.H., Li Y.L., Chiang C.L., Yang H., Yip M.C. (2013). Mechanical Properties and Tensile Fatigue of Graphene Nanoplatelets Reinforced Polymer Nanocomposites. J. Nanomater..

[114] Rajabi L., Mohammadi Z., Derakhshan A.A. (2013). Thermal Stability and Dynamic Mechanical Properties of Nano and Micron-TiO_2_ Particles Reinforced Epoxy Composites: Effect of Mixing Method. Iran. J. Chem. Eng..

[115] Shen M.Y., Liao W.Y., Wang T.Q., Lai W.M. (2021). Characteristics and Mechanical Properties of Graphene Nanoplatelets-Reinforced Epoxy Nanocomposites: Comparison of Different Dispersal Mechanisms. Sustainability.

[116] Ahn S.-H., Nam K.-W., Moon C.-K. (2013). Mechanical Properties of TiO_2_/Epoxy Resin Nanocomposites. J. Power Syst. Eng..

[117] Kumar K., Ghosh P.K., Kumar A. (2016). Improving Mechanical and Thermal Properties of TiO_2_-Epoxy Nanocomposite. Compos. B Eng..

[118] Singh S.K., Singh S., Kumar A., Jain A. (2017). Thermo-Mechanical Behavior of TiO_2_ Dispersed Epoxy Composites. Eng. Fract. Mech..

[119] Rankin S.M., Moody M.K., Naskar A.K., Bowland C.C. (2021). Enhancing Functionalities in Carbon Fiber Composites by Titanium Dioxide Nanoparticles. Compos. Sci. Technol..

[120] Yao X.F., Yeh H.Y., Zhou D., Zhang Y.H. (2006). The Structural Characterization and Properties of SiO_2_-Epoxy Nanocomposites. J. Compos. Mater..

[121] Bi Y.T., Li Z.J., Liang W. (2014). Preparation and Characterization of Epoxy/SiO_2_ Nanocomposites by Cationic Photopolymerization and Sol-Gel Process. Polym. Adv. Technol..

[122] Liang H., Yao X., Liu X., Huang Z. (2014). The Effect of Powder Bed on the Liquid Phase Sintering of α-SiC. Mater. Des. (1980–2015).

[123] Zheng W., Chen W.G., Zhao Q., Ren S.X., Fu Y.Q. (2019). Interfacial Structures and Mechanisms for Strengthening and Enhanced Conductivity of Graphene/Epoxy Nanocomposites. Polymer.

[124] Liu Q., Zhou X., Fan X., Zhu C., Yao X., Liu Z. (2012). Mechanical and Thermal Properties of Epoxy Resin Nanocomposites Reinforced with Graphene Oxide. Polym.-Plast. Technol. Eng..

[125] Adak N.C., Chhetri S., Kim N.H., Murmu N.C., Samanta P., Kuila T. (2018). Static and Dynamic Mechanical Properties of Graphene Oxide-Incorporated Woven Carbon Fiber/Epoxy Composite. J. Mater. Eng. Perform..

[126] Ashori A., Ghiyasi M., Fallah A. (2019). Glass Fiber-Reinforced Epoxy Composite with Surface-Modified Graphene Oxide: Enhancement of Interlaminar Fracture Toughness and Thermo-Mechanical Performance. Polym. Bull..

[127] Yu S., Wei D., Shi L., Ai Y., Zhang P., Wang X. (2019). Three-Dimensional Graphene/Titanium Dioxide Composite for Enhanced U(VI) Capture: Insights from Batch Experiments, XPS Spectroscopy and DFT Calculation. Environ. Pollut..

[128] Jayan J.S., Saritha A., Deeraj B.D.S., Joseph K. (2020). Graphene Oxide as a Prospective Graft in Polyethylene Glycol for Enhancing the Toughness of Epoxy Nanocomposites. Polym. Eng. Sci..

[129] Wang Q., Wang J., Lu C., Liu B., Zhang K., Li C. (2015). Influence of Graphene Oxide Additions on the Microstructure and Mechanical Strength of Cement. New Carbon Mater..

[130] Wei J., Atif R., Vo T., Inam F. (2015). Graphene Nanoplatelets in Epoxy System: Dispersion, Reaggregation, and Mechanical Properties of Nanocomposites. J. Nanomater..

[131] Lavoratti A., Zattera A.J., Amico S.C. (2018). Mechanical and Dynamic-Mechanical Properties of Silane-Treated Graphite Nanoplatelet/Epoxy Composites. J. Appl. Polym. Sci..

[132] Kamaraj M., Dodson E.A., Datta S. (2021). Thermal and Viscoelastic Behaviour of Graphene Nanoplatelets/Flax Fibre/Epoxy Composites. Plast. Rubber Compos..

[133] Ulus H. (2021). The impact of seawater aging on basalt/graphene nanoplatelet-epoxy composites: Performance evaluating by Dynamic Mechanical Analysis (DMA) and short beam shear (sbs) tests. NOHU J. Eng. Sci..

[134] German R.M., Park S.J. (2009). Handbook of Mathematical Relations in Particulate Materials Processing: Ceramics, Powder Metals, Cermets, Carbides, Hard Materials, and Minerals.

[135] MacÉ T., Vaslin-Reimann S., Ausset P., Maillé M. (2013). Characterization of Manufactured TiO_2_ Nanoparticles. J. Phys. Conf. Ser..

[136] Safdari M., Al-Haik M.S. (2018). A Review on Polymeric Nanocomposites: Effect of Hybridization and Synergy on Electrical Properties. Carbon-Based Polymer Nanocomposites for Environmental and Energy Applications.

[137] Gimenes Benega M.A., Silva W.M., Schnitzler M.C., Espanhol Andrade R.J., Ribeiro H. (2021). Improvements in Thermal and Mechanical Properties of Composites Based on Epoxy-Carbon Nanomaterials—A Brief Landscape. Polym. Test..

[138] Krishnan M.R., Alsharaeh E. (2022). A Review on Polymer Nanocomposites Based High-Performance Functional Materials. https://ssrn.com/abstract=4222854.

[139] Białkowska A., Bakar M., Kucharczyk W., Zarzyka I. (2023). Hybrid Epoxy Nanocomposites: Improvement in Mechanical Properties and Toughening Mechanisms—A Review. Polymers.

[140] Zaman I., Nor F.M., Manshoor B., Khalid A., Araby S. (2015). Influence of Interface on Epoxy/Clay Nanocomposites: 2. Mechanical and Thermal Dynamic Properties. Procedia Manuf..

[141] Das S., Halder S., Paul B., Khan N.I., Goyat M.S. (2022). Impact of Silanized Milled Graphite Nanoparticles on Thermo-Mechanical Properties of Epoxy Nanocomposite. Mater. Chem. Phys..

[142] Ahmad M.A.A., Ridzuan M.J.M., Majid M.S.A., Cheng E.M., Sulaiman M.H. (2021). Dynamic Mechanical Analysis of Graphene Nanoplatelets/Glass Reinforced Epoxy Composite. J. Phys. Conf. Ser..

[143] Baghdadi Y.N., Youssef L., Bouhadir K., Harb M., Mustapha S., Patra D., Tehrani-Bagha A.R. (2021). Thermal and Mechanical Properties of Epoxy Resin Reinforced with Modified Iron Oxide Nanoparticles. J. Appl. Polym. Sci..

[144] Karthik K., Rajamani D., Venkatesan E.P., Shajahan M.I., Rajhi A.A., Aabid A., Baig M., Saleh B. (2023). Experimental Investigation of the Mechanical Properties of Carbon/Basalt/SiC Nanoparticle/Polyester Hybrid Composite Materials. Crystals.

[145] Pan S., Feng J., Safaei B., Qin Z., Chu F., Hui D. (2022). A Comparative Experimental Study on Damping Properties of Epoxy Nanocomposite Beams Reinforced with Carbon Nanotubes and Graphene Nanoplatelets. Nanotechnol. Rev..

[146] Wu S., Sikdar P., Bhat G.S. (2023). Recent Progress in Developing Ballistic and Anti-Impact Materials: Nanotechnology and Main Approaches. Def. Technol..

[147] Shukla P., Saxena P. (2021). Polymer Nanocomposites in Sensor Applications: A Review on Present Trends and Future Scope. Chinese J. Polym. Sci. Engl. Ed..

[148] Agboola O., Fayomi O.S.I., Ayodeji A., Ayeni A.O., Alagbe E.E., Sanni S.E., Okoro E.E., Moropeng L., Sadiku R., Kupolati K.W. (2021). A Review on Polymer Nanocomposites and Their Effective Applications in Membranes and Adsorbents for Water Treatment and Gas Separation. Membranes.

[149] Jadoun S., Chauhan N.P.S., Chinnam S., Aepuru R., Sathish M., Chundawat N.S., Rahdar A. (2022). A Short Review on Conducting Polymer Nanocomposites. Biomed. Mater. Devices.

[150] Hojatzadeh S., Rahimpour F., Sharifzadeh E. (2023). A Study on the Synergetic Effects of Self/Induced Crystallization and Nanoparticles on the Mechanical Properties of Semi-Crystalline Polymer Nanocomposites: Experimental and Analytical Approaches. Iran. Polym. J. Engl. Ed..

[151] Parente J.M., Simões R., Reis P.N.B. (2022). Effect of Graphene Nanoparticles on Suspension Viscosity and Mechanical Properties of Epoxy-Based Nanocomposites. Procedia Struct. Integr..

[152] Lapčík L., Sepetcioǧlu H., Murtaja Y., Lapčíková B., Vašina M., Ovsík M., Staněk M., Gautam S. (2023). Study of Mechanical Properties of Epoxy/Graphene and Epoxy/Halloysite Nanocomposites. Nanotechnol. Rev..

[153] Di C., Yu J., Wang B., Lau A.K.T., Zhu B., Qiao K. (2019). Study of Hybrid Nanoparticles Modified Epoxy Resin Used in Filament Winding Composite. Materials.

[154] El-Masry M.M., Ramadan R., Ahmed M.K. (2020). The Effect of Adding Cobalt Ferrite Nanoparticles on the Mechanical Properties of Epoxy Resin. Results Mater..

[155] Govindaraj P., Fox B., Aitchison P., Hameed N. (2019). A Review on Graphene Polymer Nanocomposites in Harsh Operating Conditions. Ind. Eng. Chem. Res..

[156] Shameem M.M., Sasikanth S.M., Annamalai R., Raman R.G. (2021). A Brief Review on Polymer Nanocomposites and Its Applications. Mater. Today Proc..

